# Effect of ambient particulate matter pollution on disease burden globally: a systematic analysis of the global burden of disease study 2021

**DOI:** 10.1186/s12889-025-25269-5

**Published:** 2025-12-30

**Authors:** Xiaoxin Yan, Jie Han, Xiao Ding, Jianhua Jin, Liting Zhang

**Affiliations:** 1 Department of Laboratory, Changzhou wujin people’s hospital, Changzhou Medical Center, Nanjing Medical University, Changzhou, China; 2 Department of Laboratory, Changzhou wujin people’s hospital, Jiangsu University, Changzhou, China

**Keywords:** Particulate matter, Upper respiratory tract infections, Mortality, DALYs, Disease burden

## Abstract

**Background:**

Particulate matter (PM) refers to solid or liquid particles suspended in the atmosphere. These particles can be inhaled during normal respiration, leading to various respiratory diseases including upper respiratory tract infections (URTIs). We comprehensively evaluated the ambient particulate matter pollution-related disease burden.

**Methods:**

Due to the particularity of Global Burden of Disease 2021 study (GBD 2021), this study only included data on URTIs attributed to PM, and the age limit was under 5 years old. We first assessed the global and subtype-specific mortality, disability-adjusted life years (DALYs), years lived with disability (YLDs), and years of life lost (YLLs) in 2021, along with their age-standardized rates. Additionally, linear regression models were employed to analyze temporal trends in disease burden. We will calculate the corresponding estimated annual percentage change (EAPC) based on the changes in the number of deaths, DALYs and the age-standardized data of both from 1990 to 2021. Cluster analysis was used to examine regional variations in disease burden across Global Burden of Disease study regions. Finally, ARIMA and exponential smoothing (ES) models were applied to forecast disease burden over the next 25 years.

**Results:**

For indoor PM pollution, in 2021, there were 93.98 deaths per 100,000 population and 9,195.39 DALYs globally in children under 5 years of age. Females exhibited higher risks than males, and regions with a low sociodemographic index (SDI) faced elevated risks. Significant disparities in disease burden were observed across GBD regions and nations. Compared to 1990, mortality and DALYs per 100,000 population declined by 52.28% and 51.38%, respectively. ARIMA projections suggest continued declines in the absolute number of mortality and DALYs for both sexes by 2050, though age-standardized rates may increase for males while decreasing for females.

For outdoor PM pollution, 2021 recorded 22.58 deaths per 100,000 population and 2,511.13 DALYs globallyin children under 5 years of age. Females and low-middle SDI regions were at higher risk. Mortality and DALYs per 100,000 population decreased by 55.10% and 48.37% compared to 1990. ARIMA forecasts indicate further reductions in mortality, DALYs, and age-standardized rates for both sexes by 2050.

**Conclusion:**

Particulate pollutants, particularly indoor PM, pose a significant global public health threat. Tailored strategies based on national conditions are urgently needed to mitigate their impact.

## Introduction

Airborne particulate matter (PM), comprising solid or liquid particles suspended in the atmosphere, poses significant health risks upon inhalation [1]. Particles with diameters ≤ 3.5 μm can penetrate the trachea and lungs, triggering upper respiratory tract infections (URTIs) and other respiratory diseases [2]. With accelerating global industrialization, concentrations of industrial dust and combustion-derived particles have risen markedly, contributing to a substantial disease burden and emerging as a critical public health challenge worldwide [3]. The latest research shows that the burden of cardiovascular disease caused by ambient particulate matter pollution has been decreasing between 1990 and 2021, but it still poses a threat to the world [4]. Some neurodegenerative diseases are also associated with ambient particulate matter pollution [5].

PM pollution accounts for millions of premature deaths annually, reduces global life expectancy by approximately one year, and incurs trillions in socioeconomic costs [6]. Regions such as South and East Asia face particularly severe pollution levels [7]. The World Health Organization (WHO) estimates that 3% of respiratory infection-related deaths are attributable to PM exposure [8]. Primary PM sources include fossil fuel and solid fuel combustion [9]. Rapid industrialization and urban expansion in certain regions exacerbated PM pollution between 1990 and 2010, driving a surge in associated health burdens [10, 11]. Post-2010, however, many countries, notably China, implemented effective air quality controls, achieving measurable health benefits [12, 13]. Similar progress has slowed rising PM concentrations in regions like India, North Africa, the Middle East, and Central Africa [14–16]. Despite these efforts, global PM pollution remains a pressing issue, demanding sustained and innovative mitigation strategies.

While numerous studies have quantified the disease burden of PM pollution, most focus on single countries/regions or non-respiratory diseases [2, 17]. Existing research on PM-related respiratory impacts is often outdated, and few studies differentiate between indoor and outdoor PM exposure. Although recent air quality interventions have shown promise, their specific effects on disease burden remain unclear, as do future trends. To address these gaps, this study will analyzes indoor and outdoor PM-attributable disease burdens separately, with subtype-specific estimates for 2021. Then evaluates temporal trends from 1990 to 2021. Finally, we will predict burden trajectories over the next 25 years using modeling approaches.These findings aim to inform targeted public health policies for reducing PM-related health disparities across nations.

## Methods

### Overview

Due to the particularity of Global Burden of Disease 2021 study (GBD 2021), this study only included data on URTIs attributed to PM, and the age limit was under 5 years old. Annual global case counts and corresponding age-standardized rates (ASRs) were extracted from the GBD2021, which collates data from 204 countries and territories. This analysis included all indoor and outdoor particulate matter (PM)-related data from 1990 to 2021. The 204 geographical units were further stratified into 50 GBD regions based on epidemiological homogeneity and subsequently categorized into 5 tiers using the Sociodemographic Index (SDI) [18]. It is worth noting that the core indicator in GBD2021 is PM2.5, so PM in subsequent studies refers to PM2.5.

All the data in this study were extracted from data processed by GBD2021. In GBD2021, disease burden estimation was performed using DisMod-MR 2.1, a Bayesian meta-regression tool and the standard GBD modeling framework for quantifying burden by sex, age, location, and year. Data were adjusted for systematic biases via crosswalking with covariates estimated via MR-BRT. In the DisMod-MR model, excess mortality and remission rates were constrained to zero. GBD 2021 input data were derived from household surveys, vital registration systems, and other published sources [19]. There are many ways to deal with missing data, one of which is interpolation. If it is a continuous variable and the missing data is random, interpolation can be performed based on the mean or median. If data for a certain country is missing, interpolation can be performed based on data from neighboring countries or time trends. In addition, missing data can be predicted through hierarchical regression models and other methods.

### Study data

Due to the particularity of GBD2021, only the PM-attributable burden in children under 5 years of age was focused, this subgroup was analyzed separately. Only confirmed cases with clinical diagnosis were included. Data were aggregated into 50 GBD regions or 5 SDI-based groups during statistical processing.

### Statistical analysis

First, the number of deaths and DALYs attributable to outdoor particulate pollution in 2021—along with their corresponding age-standardized rates (ASRs)—were reported globally and across different subtypes (including sex, SDI, region, and country). Second, the temporal trends in the global and subtype-specific disease burden from 1990 to 2021 were statistically analyzed. The Estimated Annual Percentage Change (EAPC) was calculated using a linear regression model. Based on the EAPC values, hierarchical cluster analysis was conducted to assess the changing patterns of disease burden across different GBD regions and identify regions with similar trends. All 50 GBD regions were classified into four categories: significant increase, slight increase, stable or slight decrease, and significant decrease. Finally, future disease burdens from 2022 to 2050 were predicted using the Exponential Smoothing (ES) model and the Autoregressive Integrated Moving Average (ARIMA) model. After completing the above steps, the same data analysis procedures were applied to indoor particulate pollution data.

## Results

### Disease burden attributable to particulate matter pollution in 2021

#### Indoor particulate matter pollution

**Fig. 1:**
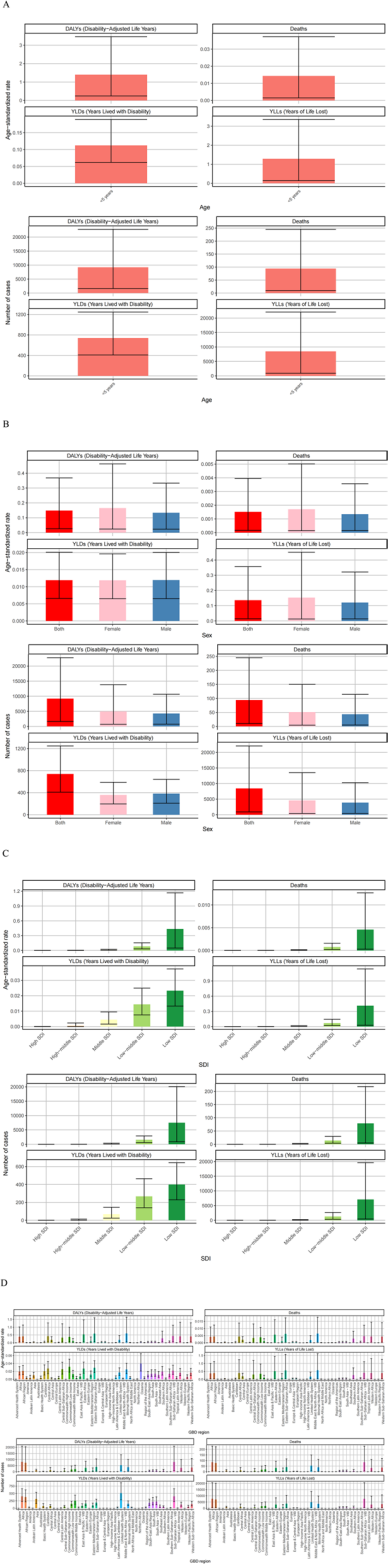
Numbers and age-standardized rates of indoor particulate matter pollution related deaths, DALYs, YLDs and YLLs for age (**A**), different sex (**B**), SDI regions (**C**) and GBD regions (**D**) in 2021

All indoor particulate matter pollution-related deaths and DALYs occurred in children under 5 years old. Deaths and DALYs in 2021 were available in Fig. 1A, indoor particulate matter pollution was associated with 93.98 deaths per 100,000 population (95% uncertainty interval [UI]: 9.91–245.34.91.34). The corresponding age-standardized mortality rate was 0.0015 per 100,000 (95% UI: 0.0002–0.0039). The attributable DALYs were 9,195.39 per 100,000 person-years (95% UI: 1,616.43-22.43.43,783.04), with an age-standardized DALY rate of 0.1485 per 100,000 (95% UI: 0.0261–0.3680).

The age-standardized mortality rate for this age group was 0.0143 per 100,000 (95% UI: 0.0015–0.0373), and the age-standardized DALY rate was 1.3971 per 100,000 (95% UI: 0.2456–3.4616).

Females showed slightly higher mortality (1.19 times that of males) and DALYs (1.17 times that of males). The corresponding age-standardized rates were 1.26 and 1.24 times higher in females than males, respectively (Fig. 1B).

Low SDI regions had the highest burden, with 78.69 deaths and 7,480.02 DALYs per 100,000 population. The age-standardized rates were also highest in these regions. Generally, lower SDI regions showed higher mortality, DALYs and age-standardized rates (Fig. 1C).

Among the 50 GBD regions, Africa had by far the highest burden, accounting for 89.83% of global deaths (84.42 per 100,000; 95% UI: 6.14–228.31.14.31) and 86.98% of DALYs (7,997.97 per 100,000; 95% UI: 968.90–20,929.82). Africa also had the highest age-standardized rates (mortality: 0.0041; DALYs: 0.3883 per 100,000) (Fig. 1D).

**Fig. 2:**
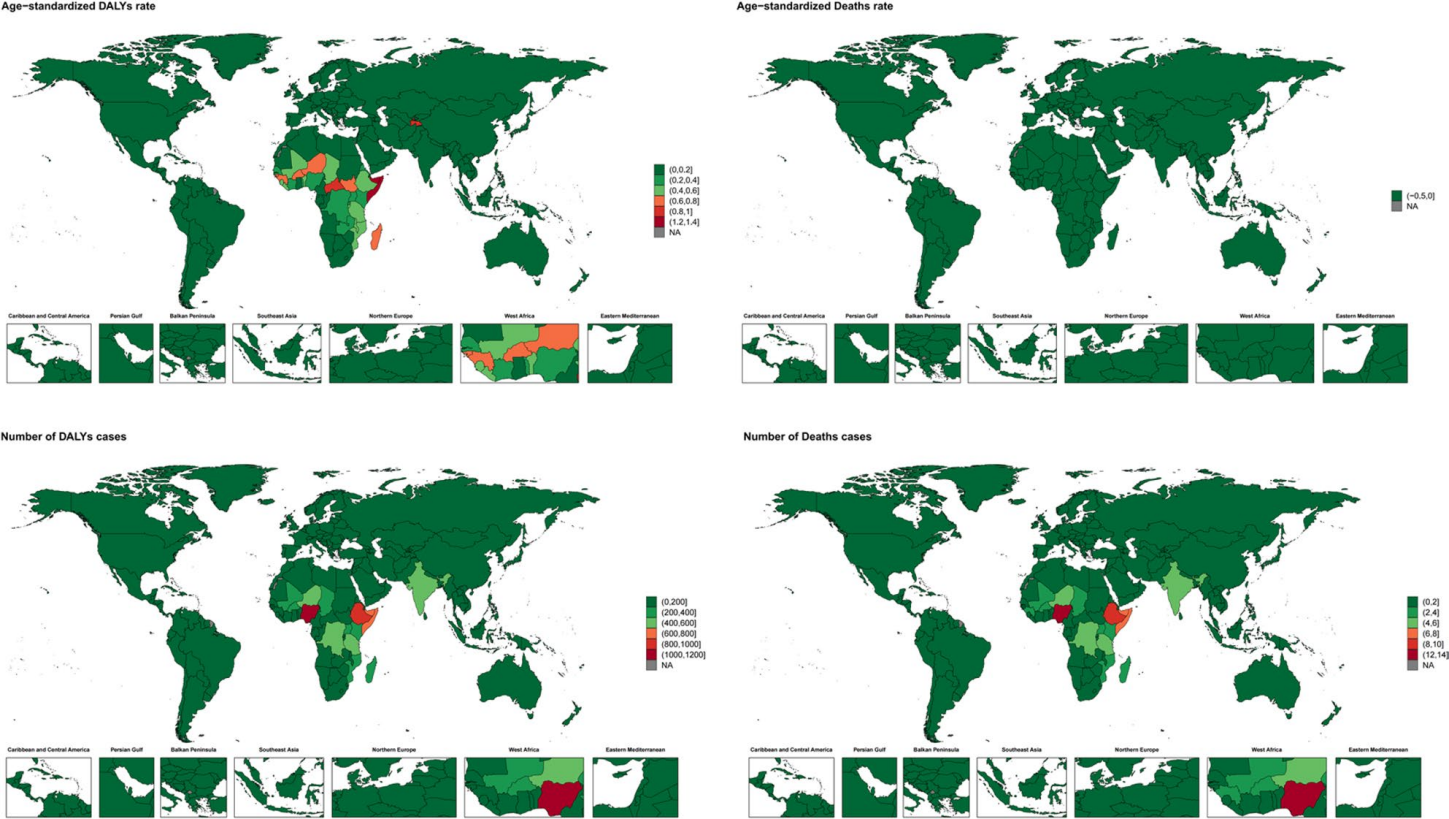
Numbers and age-standardized rates of indoor particulate matter pollution related deaths and DALYs across countries and territories in 2021

At the country level, Nigeria had the highest number of deaths (12.32 per 100,000; 95% UI: 1.55–40.11) and DALYs (1,164.96 per 100,000; 95% UI: 182.18-3.18.18,675.40), followed by Ethiopia and Somalia. Somalia had the highest age-standardized mortality rate (0.0150 per 100,000; 95% UI: 0.0002–0.0692) and DALY rate (1.3824 per 100,000; 95% UI: 0.0449–6.2730), followed by Tajikistan and the Central African Republic. The lowest burden countries were Greenland, Switzerland, Singapore and Monaco (Fig. 2).

#### Outdoor particulate matter pollution

The disease burden from outdoor particulate matter pollution followed similar patterns to indoor pollution, but with lower overall mortality, DALYs and age-standardized rates.

**Fig. 3:**
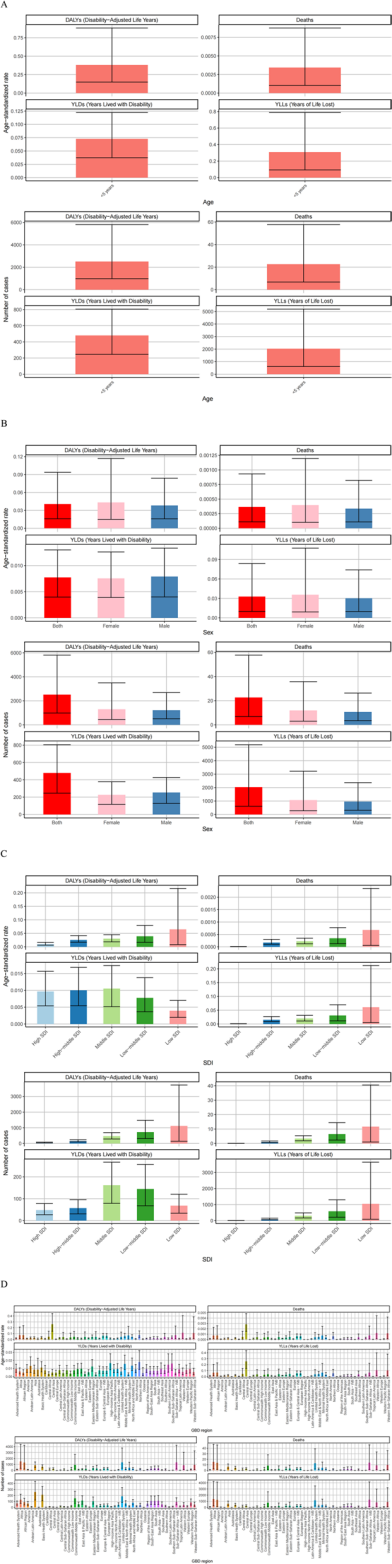
Numbers and age-standardized rates of outdoor particulate matter pollution related deaths, DALYs, YLDs and YLLs for age (**A**), different sex (**B**), SDI regions (**C**) and GBD regions (**D**) in 2021

All outdoor particulate matter pollution-related deaths and DALYs occurred in children under 5 years old. Deaths and DALYs in 2021 were available in Fig. 3A, outdoor particulate matter pollution was associated with 22.58 deaths per 100,000 population (95% UI: 6.81–57.68). The age-standardized mortality rate was 0.0004 per 100,000 (95% UI: 0.0001–0.0009). The attributable DALYs were 2,511.13 per 100,000 person-years (95% UI: 982.72-5.72.72,804.58), with an age-standardized DALY rate of 0.0406 per 100,000 (95% UI: 0.0159–0.0938).

The age-standardized mortality rate for this age group was 0.0034 per 100,000 (95% UI: 0.0010–0.0087), and the age-standardized DALY rate was 0.3815 per 100,000 (95% UI: 0.1493–0.8819).

Females showed slightly higher mortality (1.10 times that of males) and DALYs (1.06 times that of males). The corresponding age-standardized rates were 1.19 and 1.13 times higher in females than males, respectively (Fig. 3B).

Low SDI regions had the highest burden, with 11.69 deaths and 1,119.93 DALYs per 100,000 population. The age-standardized rates were also highest in these regions (Fig. 3C).

Among the 50 GBD regions, Africa had the highest number of deaths (15.22 per 100,000; 95% UI: 2.74–46.84) and DALYs (1,498.58 per 100,000; 95% UI: 329.75-4.75.75,370.35), accounting for 67.40% and 59.67% of the global totals, respectively. Asia had about half the burden of Africa. Central Asia had the highest age-standardized rates (mortality: 0.0028; DALYs: 0.2596 per 100,000) (Fig. 3D).

**Fig. 4:**
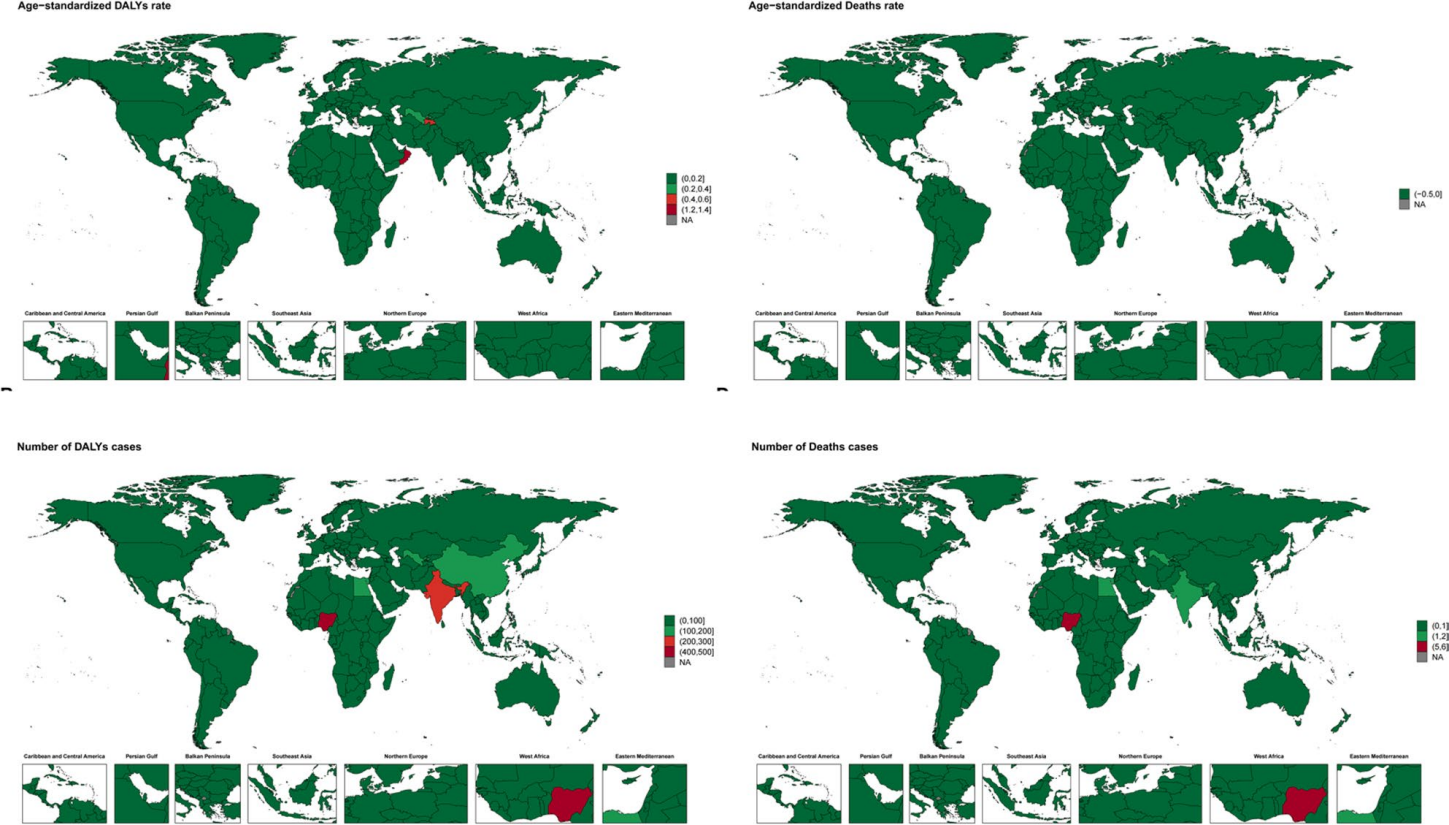
Numbers and age-standardized rates of outdoor particulate matter pollution related deaths and DALYs across countries and territories in 2021

At the country level, Nigeria had the highest number of deaths (5.13 per 100,000; 95% UI: 0.66–19.75) and DALYs (490.58 per 100,000; 95% UI: 75.97-1.97.97,811.97), followed by India and Uzbekistan. Oman had the highest age-standardized mortality rate (0.0138 per 100,000; 95% UI: 0.0055–0.0312) and DALY rate (1.2561 per 100,000; 95% UI: 0.5055–2.8309), followed by Tajikistan and Uzbekistan. The lowest burden countries were Fiji, Bermuda, Greenland and Tuvalu (Fig. 4).

### Temporal trends in disease burden attributable to particulate matter pollution, 1990–2021

#### Indoor particulate pollution

**Fig. 5:**
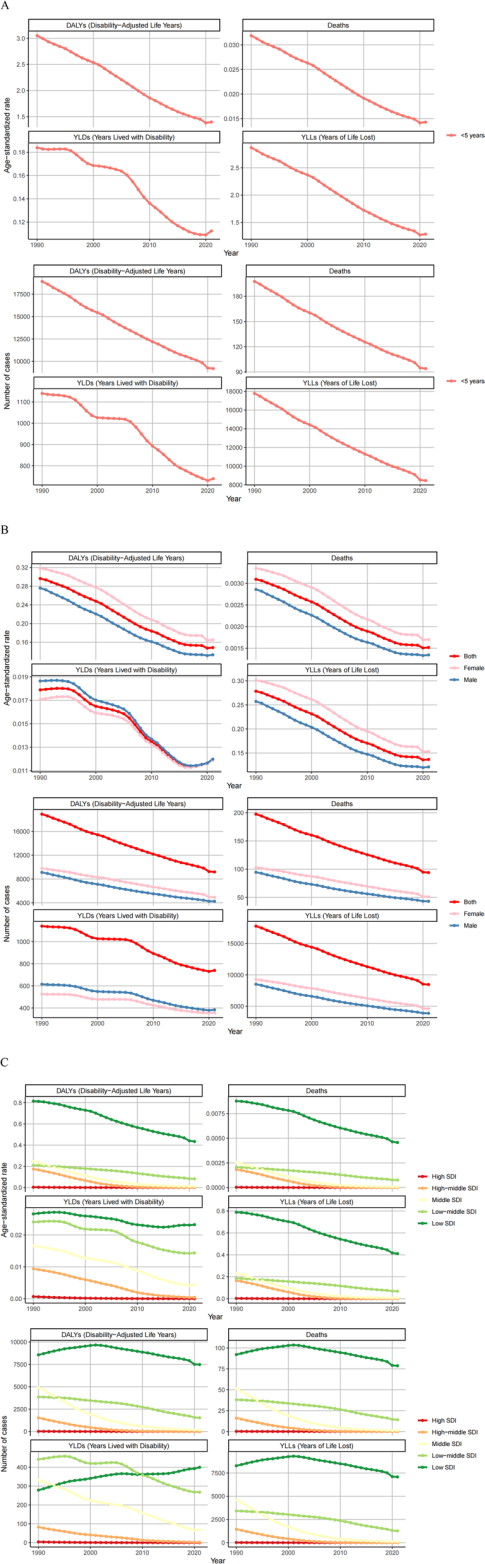
Numbers and age-standardized rates of indoor particulate matter pollution related deaths, DALYs, YLDs and YLLs between 1990 and 2021 in age (**A**), different sex (**B**) and SDI regions (**C**)

From 1990 to 2021, the global disease burden associated with indoor particulate pollution showed a consistent year-by-year decreasing trend (Fig. 5A). The number of deaths decreased from 197.55 to 93.98 per 100,000 population (a 52.43% reduction), with corresponding age-standardized mortality rates declining by 50.11%. DALYs followed the same pattern, decreasing by 51.39%, while age-standardized DALY rates fell by 49.88%.

At the gender level, both males and females exhibited trends consistent with the overall population, with all indicators showing steady declines from 1990 to 2021 (Fig. 5B).

In terms of SDI regions, high-SDI regions maintained zero cases across all indicators since 1990. Upper-middle, middle, and lower-middle SDI regions showed year-by-year decreases in deaths and DALYs. Low-SDI regions experienced increasing trends in deaths and DALYs before 2002, followed by subsequent declines.

Regarding ASRs, except for high-SDI regions which remained at zero, all other regions showed decreasing trends in mortality and DALY ASRs. By 2021, all regions except low-SDI maintained relatively low ASRs for both deaths and DALYs (Fig. 5C).

**Fig. 6:**
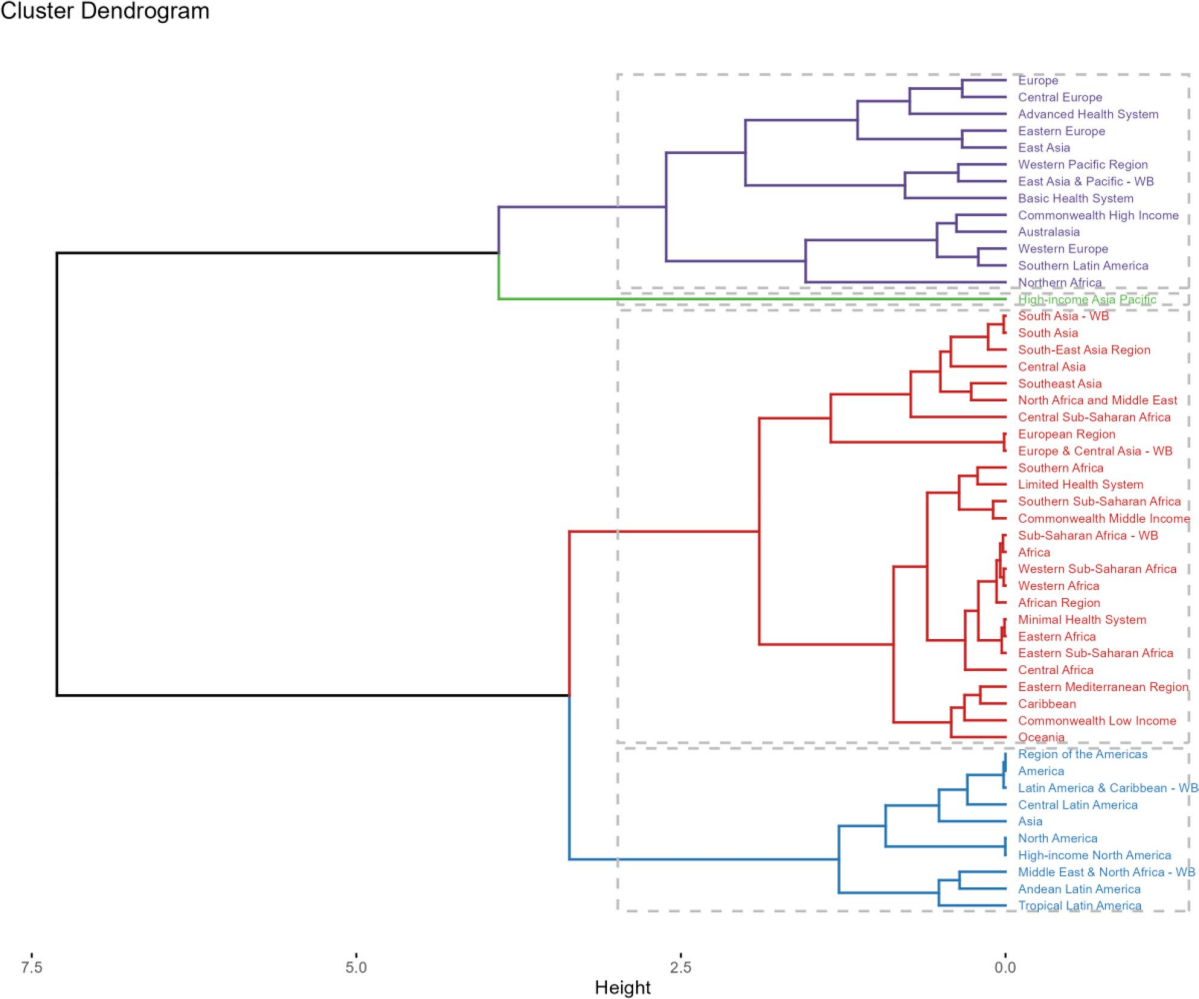
Results of cluster analysis based on the EAPC values of the indoor particulate matter pollution related age-standardized rates for deaths and DALYs from 1990 to 2021

Significant variations existed among GBD regions in indoor particulate pollution-related disease burden. Hierarchical cluster analysis was conducted to identify regions with similar burden trends. The results were shown in Fig. 6. The analysis revealed: Significant increases in mortality and DALY rates in Region of the Americas, Latin America & Caribbean-WB, and Central Latin America. Significant decreases in South Asia-WB, South-East Asia Region, North Africa and Middle East, Commonwealth Low Income, and Minimal Health System regions.

In terms of countries and regions, Afghanistan experienced the largest increase in deaths (716.85%), followed by Kuwait and Yemen. Afghanistan also showed the most significant DALY increase (368.92%), followed by Papua New Guinea and Yemen. South Korea demonstrated the most dramatic reductions (deaths: −99.99%; DALYs: −99.85%), followed by Singapore and Taiwan (China).

For ASRs, Kuwait showed the greatest increase in mortality and DALY burden (EAPC = 4.59, 95% CI 3.26–5.93), followed by Afghanistan and Northern Mariana Islands. Singapore exhibited the largest ASR reductions (EAPC=−26.78, 95% CI −27.72 to −25.81), followed by South Korea and Equatorial Guinea.

#### Outdoor particulate pollution

**Fig. 7:**
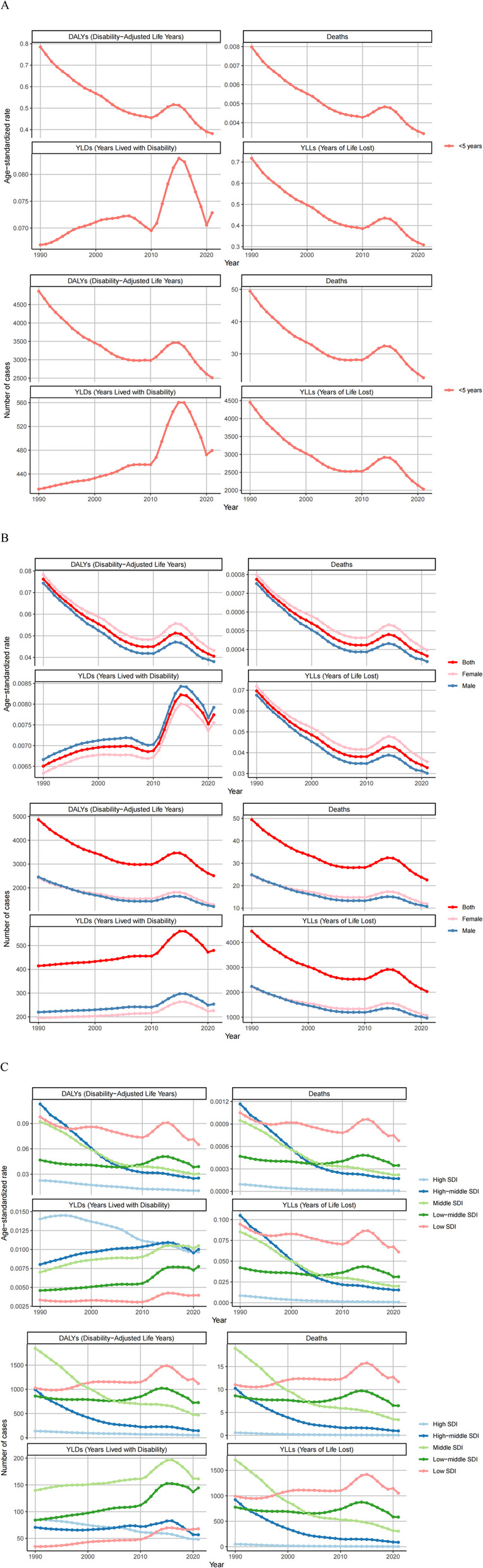
Numbers and age-standardized rates of outdoor particulate matter pollution related deaths, DALYs, YLDs and YLLs between 1990 and 2021 in age (**A**), different sex (**B**) and SDI regions (**C**)

The trends in the global disease burden associated with outdoor particulate pollution from 1990 to 2021 differed from those related to indoor particulate pollution: between 1990 and 2010, all metrics showed a year-by-year decline, but there was a slight rebound from 2010 to 2015, followed by another decline from 2016 to 2021, resulting in an overall downward trend (Fig. 7A). The global death toll from outdoor particulate pollution decreased from 49.46 cases per 100,000 people in 1990 to 22.58 cases per 100,000 in 2021, a reduction of 54.34%, with a corresponding age-standardized mortality rate decline of 52.90%. DALYs followed the same pattern, with the number of DALY cases decreasing by 48.37% and the standardized DALY rate dropping by 46.77%.

At the gender level, trends for both males and females aligned with the overall population, showing a year-by-year decline from 1990 to 2010, a slight rebound from 2010 to 2015, and another decline from 2016 to 2021, reflecting an overall downward trend (Fig. 7B).

At the SDI regional level, middle-SDI, middle-high-SDI, and high-SDI regions exhibited year-by-year declines in deaths and DALYs. In low-middle-SDI regions, case numbers decreased annually before 2006, rebounded between 2006 and 2015, and then declined again from 2016 to 2021. Low-SDI regions saw a slow increase in case numbers from 1990 to 2010, a rapid rise from 2010 to 2015, and a sharp decline from 2016 to 2021, with case numbers in 2021 nearly matching those in 1990 (Fig. 7C).

**Fig. 8:**
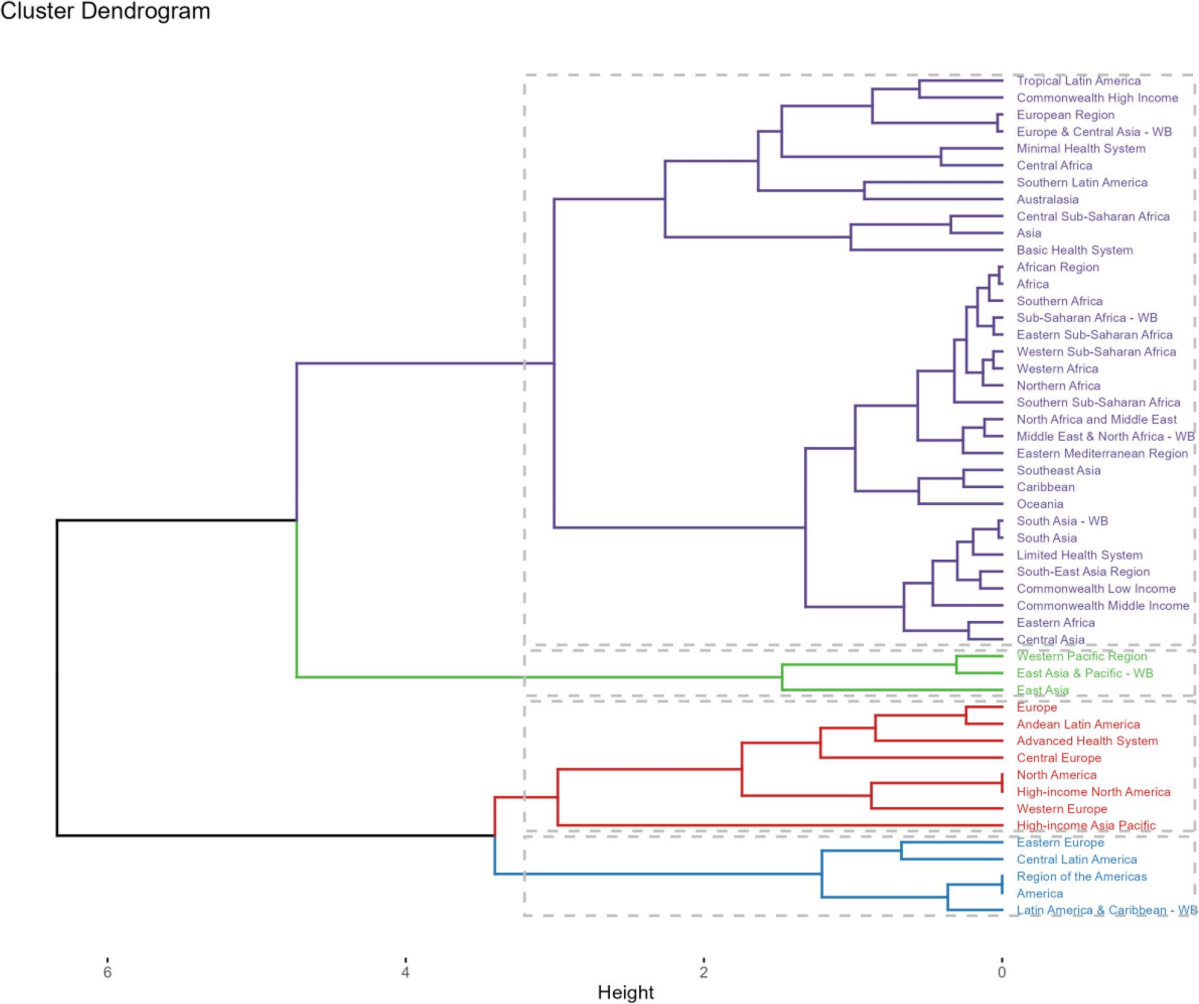
Results of cluster analysis based on the EAPC values of the outdoor particulate matter pollution related age-standardized rates for deaths and DALYs from 1990 to 2021

Among GBD regions, we also conducted a hierarchical cluster analysis on the disease burden associated with outdoor particulate pollution, with results shown in the Fig. 8. Eastern Europe, Central Latin America, the Region of the Americas, and Latin America & Caribbean - WB regions experienced significant increases in mortality and DALY rates, while Europe, Andean Latin America, Advanced Health System, Central Europe, North America, High-income North America, Western Europe, and High-income Asia Pacific regions saw significant decreases in mortality and DALY rates.

At the country and territory level, Kuwait recorded the largest increase in death cases from 1990 to 2021, at 5592.95%, followed by Qatar and Afghanistan. Afghanistan showed the most pronounced rise in DALY cases, at 368.93%, followed by Qatar and Sudan. Mexico exhibited the most significant decline in case numbers (deaths: −98.10%; DALYs: −97.05%), followed by Lithuania and Latvia. For age-standardized rates (ASRs), Kuwait saw the largest increase in death and DALY burdens from 1990 to 2021 [EAPC = 15.71, 95% confidence interval (CI) 13.88–17.58], followed by Sudan and Yemen. The greatest decline was observed in Singapore (EAPC = −13.78, 95% CI −14.85 to −12.69), followed by Taiwan (China) and Japan.

### Projected results from 2021 to 2050

#### Indoor particulate matter pollution

**Fig. 9:**
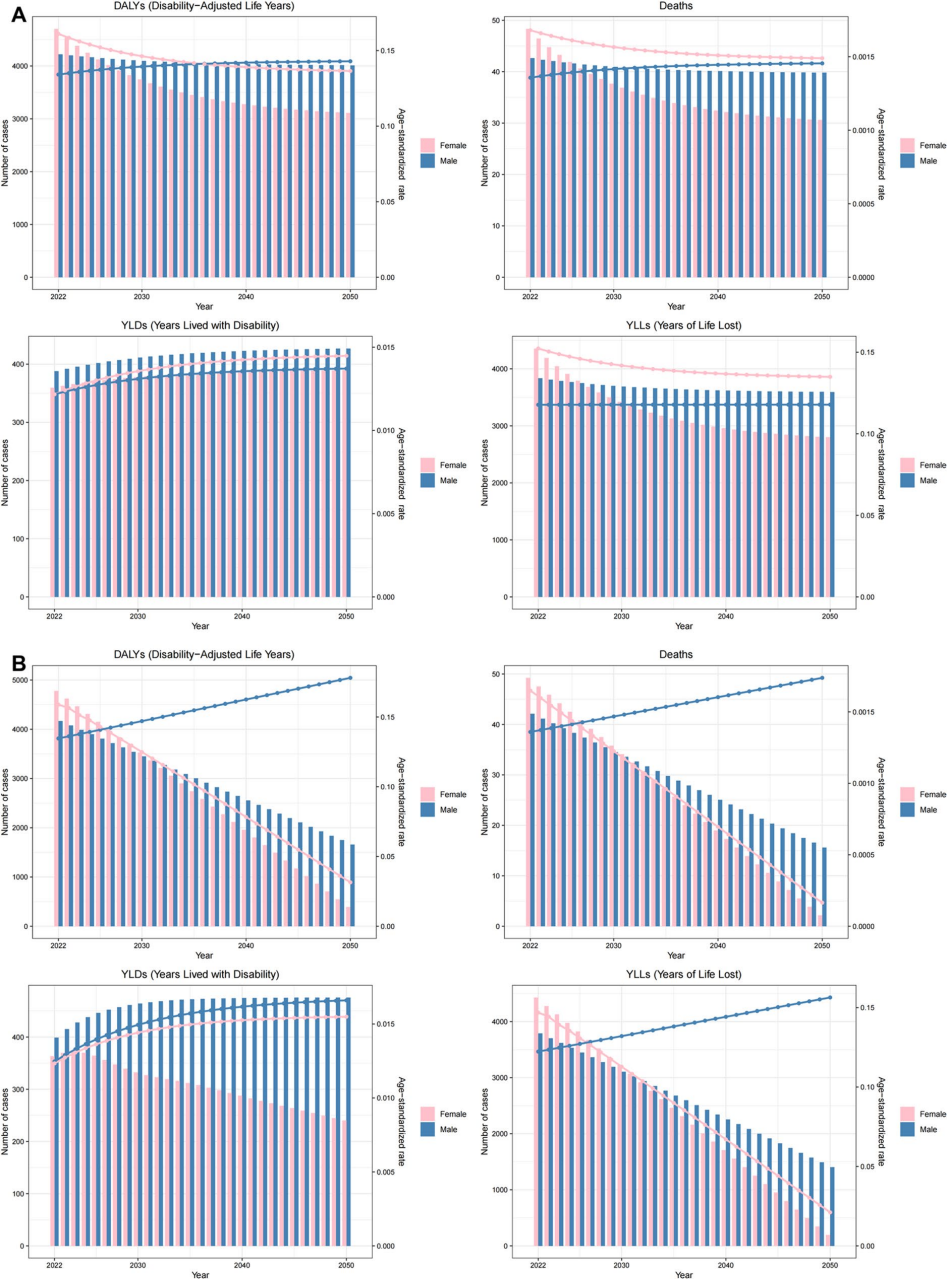
The predicted results in the indoor particulate matter pollution related numbers and age-standardized rates of deaths and DALYs by sex globally from 2021 to 2050 of the ES model (**A**) and ARIMA model (**B**)

The exponential smoothing (ES) model predicts a declining trend in both mortality and disability-adjusted life years (DALYs) attributable to indoor particulate matter pollution among females from 2021 to 2050, while males show a stable pattern with marginal decreases. Age-standardized rates (ASRs) for female mortality and DALYs demonstrate consistent annual reductions during this period, whereas male ASRs exhibit a slight upward trend. These projections are corroborated by corresponding ARIMA model results, confirming the robustness of our predictions (Fig. 9).

#### Outdoor particulate matter pollution

**Fig. 10:**
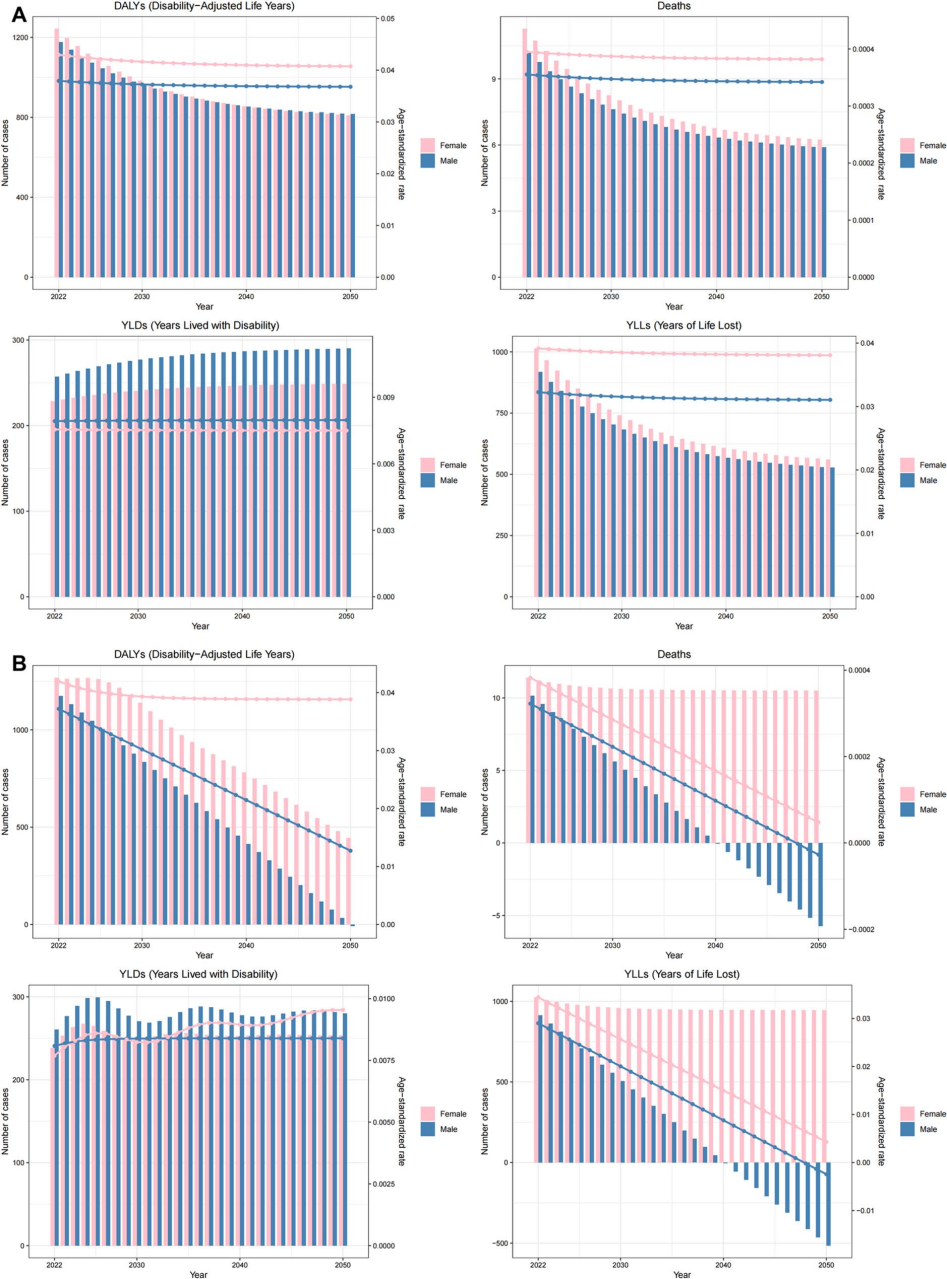
The predicted results in the outdoor particulate matter pollution related numbers and age-standardized rates of deaths and DALYs by sex globally from 2021 to 2050 of the ES model (**A**) and ARIMA model (**B**)

ES model projections indicate decreasing mortality and DALYs related to outdoor particulate pollution for both genders between 2021 and 2050, with corresponding ASRs showing progressive annual declines. This trend is similarly reflected in ARIMA model outputs (Fig. 10).

## Discussion

This study provides a comprehensive global assessment and quantification of disease burden attributable to particulate matter pollution, with projections of future trends. Our analysis reveals that in 2021, both indoor and outdoor particulate pollution imposed substantial disease burdens worldwide, particularly among children under five years old, with significant disparities observed across genders, SDI regions, GBD regions, and nations. Notably, the disease burden associated with indoor particulate pollution consistently exceeded that of outdoor pollution. Temporally, indoor particulate-related burdens showed continuous reduction from 1990 to 2023, while outdoor pollution-related burdens experienced a resurgence after 2010 before declining again post-2016. Model projections suggest that over the next 25 years, all particulate-related disease burdens will decrease except for male ASRs related to indoor pollution, which are predicted to rise gradually.

Existing literature has examined particulate pollution-related disease burdens from various perspectives [2, 17], though most studies have focused on individual countries or regions, with limited global-scale analyses [20]. Even among global studies, the emphasis has typically been on specific disease categories, such as respiratory [21] or cardiovascular diseases [22], or renal conditions [23]. There remains a paucity of comprehensive global assessments of overall particulate pollution disease burden.

Previous research methodologies, particularly the American Cancer Society Cancer Prevention Study II(CPS-II) cohort, while valuable, may have limited generalizability due to their localized sampling [24]. In contrast, the GBD 2021 study incorporates diverse data sources including household surveys and vital statistics across multiple nations, yielding more robust estimates [19]. Cohen et al.‘s analysis of GBD 2015 data revealed that particulate pollution caused 4.2 million excess deaths and 103.1 million DALYs in 2015 [25]. However, these findings require updating. Our current study provides a thorough evaluation of global indoor and outdoor particulate pollution burdens, consolidating evidence that these environmental factors impose substantial health impacts.

The observed decline in standardized particulate pollution burdens from 1990 to 2021 likely reflects global improvements in combustion technologies and transitions to cleaner energy [26, 27]. Developing nations, particularly China, have contributed significantly through industrial restructuring toward high-value, low-pollution sectors [28]. Future strategies should emphasize clean fuel adoption in high-income regions and improved cookstove programs in low-income areas interventions proven effective for burden reduction [29, 30]. However, the disease burden of outdoor particulate matter pollution has experienced a significant rebound since 2010. The extreme high temperatures experienced by the Earth in 2016 and the strong El Niño phenomenon in 2015–2016 are likely to have contributed to the disease burden of outdoor particulate matter pollution since 2010.

Our findings demonstrate greater particulate-related disease burdens among females, consistent with studies showing heightened female susceptibility to respiratory symptoms and inflammatory responses [31]. Notably, particulate exposure correlates with fatal coronary heart disease risk in women but not men, even after controlling for confounders like tobacco and alcohol [32]. Potential explanations include sex hormone-mediated differential pulmonary responses to pollutants and ovarian reserve depletion through accelerated follicular recruitment and apoptosis [33–35]. However, some studies report male predominance in pollution-related COPD mortality [36], suggesting complex interactions with lifestyle and occupational factors [37]. The precise mechanisms underlying gender disparities remain unclear and warrant further investigation.

Significant cross-national burden disparities, previously documented [25], likely stem from environmental, preventive care, and developmental differences. For instance, India’s persistently high particulate levels reflect population growth, vehicular emissions, poor-quality fuels, and inadequate environmental regulations [38]. The highest burdens in low-middle SDI regions underscore the development-environment tradeoff in developing nations. The rapid decline in disease burden in low SDI countries after 2002 may be due to the signing of important international environmental agreements such as the Kyoto Protocol. Low SDI countries began to pay attention to the governance of environmental pollution, which helped these regions to rapidly reduce the disease burden.

Projections for 2025–2050 indicate declining mortality and DALYs across genders, with generally decreasing ASRs, continuing 1990–2021 trends. Global environmental consensus and clean energy policies [39], including electric vehicle adoption [40], support this trajectory. However, demographic shifts, risk behaviors, and socioeconomic development may challenge pollution control, necessitating additional evidence-based policies.

The findings from GBD 2021 indicate that while the disease burden attributable to particulate matter pollution is projected to decline in the coming years, it remains a significant public health threat. This underscores the need for sustained and targeted interventions, such as stricter air quality regulations, promotion of clean energy alternatives, and urban planning strategies to reduce exposure. Additionally, cross-sector collaboration is critical to mitigate emissions at the source. Even with progress, continued investment in research and adaptive policies will be essential to address residual risks and emerging challenges, such as climate change interactions with air pollution.

Due to the reliance on data from the GBD database, this study has several limitations. A significant challenge is the lack of detailed data from counties, provinces, and states [41]. GBD2021 provides data at the local level for some countries, such as China, but the overall granularity is not sufficient for local-level analysis in all countries. Additionally, GBD2021 limits the age of URTIs deaths and DALYs attributed to PM to “children under 5 years old”, which makes this study lack analysis on an age scale. In addition, GBD2021 only includes confirmed cases with clinical diagnosis, but in some low-income countries, child deaths are often caused by multiple factors, and diagnostic errors may affect the authenticity of data in these countries. Finally, the predictions in this study assume that other factors remain constant over the next 25 years. It should be noted, however, that some variables may change.

## Conclusion

In summary, both indoor and outdoor particulate pollution impose a substantial disease burden worldwide, particularly in low SDI regions. In other regions, low-middle SDI areas still have some disease burden, while other areas have low disease burden. Additionally, our study provides evidence that women and children are high-risk groups. We also found that over the next 25 years, the number of outdoor cases will gradually decline before stabilizing, indicating that pollution control measures have achieved partial success. However, the ASR of indoor particulate pollution-related cases will increase for male and continue to decrease for female, which means that men will face a higher threat of disease. Particulate pollution remains a critical public health issue that requires further attention. More stringent mitigation and adaptation strategies should be implemented and designed to protect individuals and control particulate pollution. For policymakers, these data underscore the need to maintain and strengthen air quality regulations while prioritizing areas where progress is lagging. Health professionals can use these projections to advocate for targeted clinical interventions and public awareness campaigns. Community leaders should use localized burden estimates to drive equitable solutions while holding authorities accountable. The study’s global trends also highlight the importance of cross-border collaboration and long-term monitoring to ensure gains are not reversed.

## Data Availability

No datasets were generated or analysed during the current study.
